# Supplementary material for: Anisotropic fluid with phototunable dielectric permittivity
Source: Nat Commun. 2022 Mar 3;13:1142. doi: 10.1038/s41467-022-28763-1 (PMC8894468; doi:10.1038/s41467-022-28763-1)
Supplement: Supplementary file 1 — Supplementary Information [file 41467_2022_28763_MOESM1_ESM.pdf]

Supplementary Information

Anisotropic fluid with phototunable dielectric permittivity

Hiroya Nishikawa*, Koki Sano* and Fumito Araoka*

*To whom correspondence should be addressed.

E-mail: hiroya.nishikawa@riken.jp (H.N.), koki.sano@riken.jp (K.S.), fumito.araoka@riken.jp (F.A.)

Table of contents

Methods

1. **General and materials** S2
2. **Synthesis and characterization of Azo-F** S3

Supplementary Notes (Supplementary Notes 1–4) S6

Supplementary Figures (Supplementary Figs. 1–20) S10

Supplementary Tables (Supplementary Tables 1–2) S31

Supplementary Audio S34

Supplementary References S35

Methods

1. General and materials

General: Analytical thin layer chromatography (TLC) was performed on silica gel layer glass plate Merck 60 F254 and visualized by UV irradiation (254 nm). Column chromatography was performed on a Biotage Isolera^{TM} Prime flash system (Biotage) using Biotage SNAP Ultra (25 g; particle size 25 μm; HP-spherical silica) column cartridge. ^{1}H and ^{13}C nuclear magnetic resonance (NMR) spectra were recorded on Ascend 600 (600 MHz, BRUKER) operating at 600.00 MHz and 150.00 MHz for ^{1}H and ^{13}C NMR, respectively, using the TMS (trimethylsilane) as an internal standard for ^{1}H NMR and the deuterated solvent for ^{13}C NMR. The absolute values of the coupling constants are given in Hz, regardless of their signs. Signal multiplicities were abbreviated by s (singlet), d (doublet), t (triplet), q (quartet), quint (quintet), sext (sextet), dd (double–doublet), respectively. Electron ionization mass spectrometry (EIMS) and High resolution EIMS were performed on JMS-700 (JEOL) at Kyushu University (IMCE). The UV-Vis spectra were recorded using a UV-Vis-NIR spectrophotometer (V-670, JASCO) using a 1 cm-thick quartz cuvette or quartz sandwich cell. Polarized optical microscopy were performed on a polarizing microscope (Eclipse LV100 POL, Nikon) with controlling the temperature using a temperature controller and a hot stage (mK2000, INSTEC). Unless otherwise noted, the sample temperature was controlled using INSTEC model mK2000 temperature controller. Differential scanning calorimetry (DSC) was performed on a calorimeter (DSC30, Mettler-Toledo). Cooling and heating profiles were recorded and analyzed using the Mettler-Toledo STAR^{e} software system. X-ray diffraction (XRD) measurements were carried out by using a NANOPIX 3.5m system (Rigaku) with a detector (HyPix-6000, Rigaku). Dielectric relaxation spectroscopy was performed ranging between 1 Hz and 1 MHz using an impedance/gain-phase analyzer (SI 1260, Solartron Metrology) and a dielectric interface (SI 1296, Solartron Metrology). Prior to starting measurement of the LC sample, the capacitance of the empty cell was determined. Magnetic orientation of liquid-crystalline molecules was performed by using a 9-T superconducting magnet (Cryogenic). Light irradiation was performed on a LED driver (DC2200, Thorlabs) equipped with a green LED (525 nm, SOLIS-525C, Thorlabs, I = 180 mW cm^{-2}) or a blue LED (415 nm, M415LP1, Thorlabs, I = 7.0 mW cm^{-2}) heads.

Materials: All of reagents and solvents were purchased from Kanto Chemical Co., Inc., Tokyo Chemical Industry Co., Ltd., FUJIFILM Wako Pure Chemical Corporation and Combi-Blocks, Inc. and used without further purification. A host liquid crystal (DIO) and photo-triggers (Azo-H, Azo-Me, and Azo-F) were synthesized in our laboratory. Azo-H and Azo-Me were prepared according to refs. 1 and 2, respectively.

2. Synthesis and Characterization of Azo-F

The photo-trigger, Azo-F (**1**) was synthesized as follows:

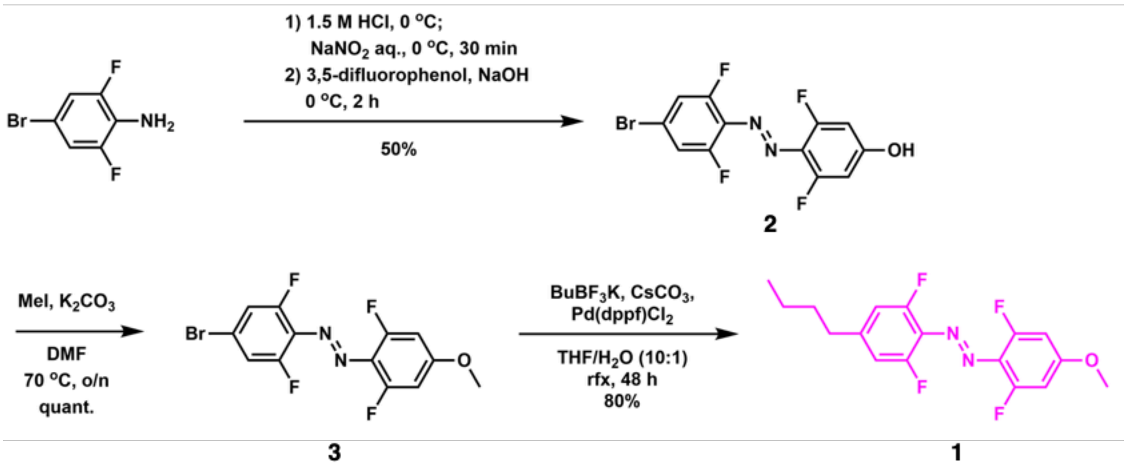

2.1. (*E*)-4-((4-bromo-2,6-difluorophenyl)diazenyl)-3,5-difluorophenol (2).

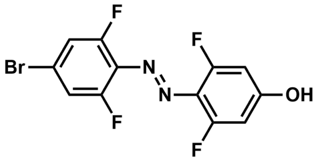

An precooled aqueous solution of 5% $NaNO_2$ (1.46 g, 21.2 mmol) was added dropwise into a solution of 4-bromo-2,6-dimethylaniline (2.00 g, 9.60 mmol) in 1.5 M HCl (14.1 mL) at 0 °C followed by stirring for 30 min keeping 0 °C , the mixture was added dropwise to a solution of phenol in an aqueous solution of NaOH (1.27 g, 31.7 mmol) at 0 °C to yield the orange precipitate immediately. After stirring for 2 h, the resulting mixture was extracted by Et_2O, washed with water, dried over anhydrous Na_2SO_4, evaporated in vacuo and separated by chromatography (SiO_2, 0–2% MeOH/DCM) to afford title compound **(2)** as an orange solid (1.68 g, 50%). R_f = 0.16 (DCM); ^{1}H NMR (600 MHz, TMS, DMSO-*d6*): δ = 7.410 (d, *J* = 8.4 Hz), 6.542 (d, *J* = 11.4 Hz) ppm; ^{13}C NMR (150 MHz, Methanol-*d4*): δ = 164.09 (t), 159.12 (dd), 156.54(dd), 132.43 (t), 126.10 (t),

123.33 (t), 117.59 (dd), 101.10 (dd) ppm; HRMS (EI, *m*/*z*, $[M]^+$) Calcd for $C_{12}H_5BrF_4N_2O$: 347.9521; found: 347.9519.

2.2. (*E*)-1-(4-bromo-2,6-difluorophenyl)-2-(2,6-difluoro-4-methoxyphenyl)diazene (3).

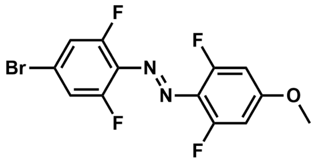

Iodomethane (0.21 mL, 3.30 mmol) was added into a solution of compound (**2**) (0.30 g, 0.70 mmol) and K_2CO_3 (0.46 g, 3.30 mmol) in DMF (3.3 mL, 0.20 M). After stirring overnight at 70 °C, the resulting mixture was cool to room temperature and then distilled water was poured. The mixture was extracted by Et_2O, washed with water, dried over anhydrous Na_2SO_4, evaporated in vacuo and separated by chromatography (SiO_2, Hexane → DCM) to afford title compound **(3)** as an orange solid (0.20 g, quant.). R_f = 0.84 (DCM), 0.77 (Hexane : DCM = 5 : 5); ^{1}H NMR (600 MHz, TMS, Chloroform-*d*): δ = 7.239 (d, *J* = 8.4 Hz), 6.596 (d, *J* = 11.4 Hz), 3.882 (s) ppm; ^{13}C NMR (150 MHz, TMS, Chloroform-*d*): δ = 162.98 (t), 157.56 (dd), 155.38 (dd), 131.16 (t), 126.03 (t), 122.63 (t), 107.76 (d), 99.98 (d), 56.22 (q) ppm; HRMS (EI, *m*/*z*, $[M]^+$) Calcd for for $C_{13}H_7BrF_4N_2O$: 361.9678; found: 361.9679.

2.3. Synthesis of (*E*)-1-(4-butyl-2,6-difluorophenyl)-2-(2,6-difluoro-4-methoxyphenyl)diazene (1).

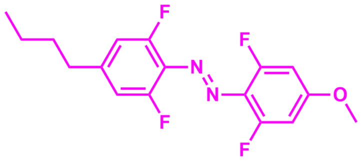

In a 20 mL two-neck flask were added compound **(3)** (240 mg, 0.66 mmol), $PdCl_2$(dppf) (48.6 mg, 9 mol%), $CsCO_3$ (646 mg, 1.98 mmol) and $BuBF_3K$ (163 mg, 0.99 mmol) and then Ar gas was purged. A solution of THF/H_2O (10:1) (6.60 mL, 0.10 M) was added into the sealed flask via syringe and then the mixture was refluxed for 48 hours. After finishing reaction, the mixture was cooled to room temperature. The resulting mixture was extracted by Et_2O, washed with water, dried over anhydrous Na_2SO_4, evaporated in vacuo. The crude was passed through a double layer silica gel column (Florisil, 1 cm and SiO_2, 1 cm, eluent: DCM) to remove residual Pd. The obtained crude

was separated by chromatography (SiO_2, Hexane → Hexane : DCM = 7 : 3) and purified by recrystallization from hexane (three times) to afford title compound (**1**) as an orange needle (157 mg, 80% yield). R_f = 0.83 (Hexane : DCM = 5 : 5); ^{1}H NMR (600 MHz, TMS, Chloroform-*d*): δ = 6.854 (d, *J* = 10.2 Hz), 6.586 (d, *J* = 10.8 Hz), 3.869 (s), 2.634 (t, *J* = 7.5 Hz), 1.632 (quin, *J* = 7.5 Hz), 1.368 (sext, *J* = 7.5 Hz), 0.942 (t, *J* = 7.5 Hz) ppm; ^{13}C NMR (150 MHz, Chloroform-*d*): δ = 162.25 (t), 157.34 (dd), 155.52 (dd), 147.53 (t), 129.78 (t), 126.15 (t), 112.30 (dd), 98.88 (dd), 56.14 (s), 35.52 (s), 32.70 (s), 22.14 (s), 13.83 (s) ppm; HRMS (EI, *m*/*z*, $[M]^+$) Calcd for $C_{17}H_{16}F_4N_2O$: 340.1199; found: 340.1197.

Supplementary Notes (Supplementary Notes 1–4)

Supplementary Note 1 | Polarized optical microscopy and dielectric spectroscopy under a magnetic field

The polarized optical microscopy and dielectric spectroscopy were performed under a magnetic field ranging from 9 to 0 T in the N_F phase (60 °C) of DIO, which was injected into a silanized cell (gap: 21.4 μm). The measurement system was shown in Supplementary Fig. 7a,b. For system A, a magnetic field is applied vertical to the LC cell so that the parallel component of dielectric permittivity along the director ($\varepsilon_{\parallel}$) can be evaluated. On the other hand, by applying magnetic field vertical to the LC cell (system B), one can evaluate the variation of vertical component of dielectric permittivity ($\varepsilon_{\perp}$). For both of system, the corresponding polarized optical microscopic image changes were observed simultaneously with dielectric spectroscopy. In these experiments, we used a handmade ITO glass hot-stage (see left image in the panel (a) in Supplementary Fig. 7). The measurement temperature was controlled and monitored using a DC power supply and a handmade temperature monitor, respectively. Firstly, after rising temperature to 120 °C (N phase), a magnetic field was gradually increased to 9 T, at which a dark field was observed in POM under crossed polarizers for both of system (not shown here). It indicates that the nematic director reorients along the corresponding magnetic field, realizing out-of-plane and in-plane alignment in system A and B, respectively. After temperature was decreased to 60 °C (N_F phase) with keeping a magnetic field of 9 T, polarized optical microscopy and dielectric spectroscopy were performed in various magnetic fields (9 to 0 T). In system A (9 T), a dark field due to out-of-plane alignment in the N phase changed to the polydomain structure with a domain size of the order of a few micrometers in the N_F phase at 60 °C (Supplementary Fig. 7c). As the decrease of a magnetic field, the coarsening of domain size started, the wavy structure, which was maybe due to Helfrich deformation, was generated [3]. Finally, the coarsening of the wavy domain ceased, the characteristic texture of the N_F phase was observed at B = 0 T. In this process, the magnitude of dielectric permittivity (≈ 13,000) slightly increased and the relaxation peak (f = 1 kHz) shifted to the high frequency regime (log$f$ = 3.8) (Supplementary Fig. 7e,f). By contrast, in system B, the in-plane orientation remained during phase transition from the N to N_F phase at B = 9 T (Supplementary Fig. 7d). Thus, this confirmed that the in-plane alignment along the direction of the applied magnetic field occurred by the strong magnetic field parallel to the LC cell without rubbing treatment. With

decreasing a magnetic field, the birefringence color and the tiny domain texture were observed, the peak position still remained at log*f* = 3.8 (Supplementary Fig. 7d,g). Thus, in the N_F phase, the degenerate in-plane alignment is preferable in the absence of a magnetic field because of exact-match DR properties at B = 0 T (Supplementary Fig. 7e,g). Noteworthy, the parallel component of the dielectric permittivity showed the gigantic permittivity (≈ 13,000), which accorded closely with the vertical component of one, irrespective of the magnitude of *M*-filed (Supplementary Fig. 7g,h). Therefore, the gigantic dielectric nature owing to the polar collective motion in the N_F phase is not macroscopically anisotropic. This point is significantly unique because the gigantic dielectric permittivity and capacitance based on the large polarization of the N_F phase can be accepted without any alignment treatment.

Supplementary Note 2 | Photoisomerization of *ortho*-fluoroazobenzenes

The commonly-known azobenzenes have two distinct absorption bands around 365 nm (UV, π→π* transition) and 450 nm (blue, n→π* transition) in the ground *trans*-isomer state, and the former is usually irradiated to induce the *trans*-to-*cis* photoisomerization. As for the back reaction process, i.e., the *cis*-to-*trans* photoisomerization, the n→π* transition of the *cis*-isomer is utilized with the blue light irradiation. However, this means that the absorption band at the blue region is in fact the superimposed n→π* transition bands of *trans*- and *cis*-isomers. Thus, if the n→π* transition bands of the *trans*- and *cis*-isomers can be effectively separated in the visible region, we are able to utilize the *trans* n→π* transition to induce the *trans*-to-*cis* photoisomerization and don't have to rely on the harmful UV light. In the case of Azo-F, the n→π* band of the *cis*-isomer is blue-shifted due to the lower energy of the n-orbital of the *cis*-isomer [because the fluorine substituents in the *ortho*-positions (i.e. nearby σ-electron-withdrawing groups) effectively reduce the n-electron density], resulting in the effective separation of the n→π* absorption bands as shown in Fig. 1h,i, and then the green light (above 500 nm) becomes accessible to the *trans*-to-*cis* isomerization through the n→π* transition of the *trans*-isomer. Bleǵer et al. discussed such a band separation process based on the molecular orbital (MO) theory [Bleǵer, D. et al. *J. Am. Chem. Soc.* **134**, 20597–20600 (2012)].

Supplementary Note 3 | Thermal properties of [Azo-F] (0–8 wt%)

The POM images and the DSC curves for [Azo-F] (0–8 wt%) are summarized in Supplementary Fig.

10. For a series of [Azo-F], in the M phase, the sandy texture was observed whereas the specific defect lines appeared in the N_F phase (Supplementary Fig. 10a). The M–N_F phase transition point of a series [Azo-F] was shifted to the lower temperature side with increasing a concentration of a photo-trigger, Azo-F (Supplementary Fig. 10b). As listed in Supplementary Table 2, it should be noted that the enthalpy, $\Delta H_{M\text{–}NF}$, of [Azo-F] (0 wt%, *i.e.,* the pure DIO) and [Azo-F] (1–4 wt%) are quite similar, indicating the doped Azo-F does not disturb the order parameter and polar order of intrinsic N_F phase. Thus, [Azo-F] (1–4 wt%) successfully inherit the M–N_F phase transition and its nature of the pure DIO. On contrary, for LC blends with high concentration of Azo-F (6 and 8 wt%), the M–N_F phase transition was also confirmed; however, the corresponding $\Delta H_{M\text{–}NF}$ was lower than that of estimated one ($\Delta H_{estimated}$), which is considering a volume function of Azo-F against the host DIO. It suggests that the high concentration of Azo-F may have a detrimental effect on the nature of N_F phase. In fact, the dielectric permittivity was significantly decreased for [Azo-F] (over 6 wt%) (Fig. 2b in the main text).

Supplementary Note 4 | Oscillator circuit.

The 555 Timer IC is one of the commonly used ICs in thousands of applications since it can generate accurate timing pulses. This IC has mainly two operating modes, i.e., monostable and astable mode. In the monostable mode, time delay of the pulse can be precisely controlled whereas the frequency and duty cycle can be controlled in the astable mode. Especially, the astable mode can generate highly accurate free running waveforms whose output frequency is adjustable by using the external *RC* network. Therefore, we adopted the astable mode. The measurement circuit is shown in Supplementary Fig. 17a. In the astable oscillator circuit, the condenser charges up to $2/3V_s$ (the upper comparator limit) whereas discharging it down to $1/3V_s$ (the upper comparator limit) (Supplementary Fig. 17b). The charge (t_1) and discharge (t_2) times required during each cycle of the output are given as follows:

$$t_1 = \ln 2(R_A + R_B)C \quad (1)$$

and

$$t_2 = \ln 2(R_B)C, \quad (2)$$

respectively. W here, *R* and *C* are resistor (Ω) and capacitor (F), respectively. Besides, in the astable mode, these individual times, that is, the frequency (*f*) is independent on the supply voltage. Thus, the duration of one full timing cycle (T) is represented as:

$$T = t_1 + t_2 = \ln 2(R_A + 2R_B)C. \quad (3)$$

Hence, the output frequency (f) for the total cycle timing is expressed below:

$$f = \frac{1}{T} = \frac{1.44}{(R_A + 2R_B)C}, \quad (4)$$

where R represents resistors, while C is capacitance of the photo-variable capacitor based on [**Azo-F**]. The photo-tunable range of the output frequency was adjusted so as to generate the sound waves at the frequencies between 20 Hz (infrasound) and 20 kHz (ultrasound) which is the common hearable range of human. Thus, on the basis of Eq. 4, we chose R_A = 200 (Ω) and R_B = 20 (kΩ) for the resistors in the oscillator circuit. In fact, the obtained tunable range (100 Hz to 8.5 kHz) was slightly different from that (120 Hz to 6.5 kHz) estimated from the capacitance values obtained with the impedance analyzer. This mismatch would be attributed to nonlinearity in the oscillator circuit caused by the non-ideal response in the sample, such as inherent/photo-induced ionic conduction, field-/light-induced molecular reorientation, photofluidization, etc. It is important to note that, despite this fact, the order of magnitude of the tunable range was still acceptable.

Supplementary Figures (Supplementary Figs. 1–20)

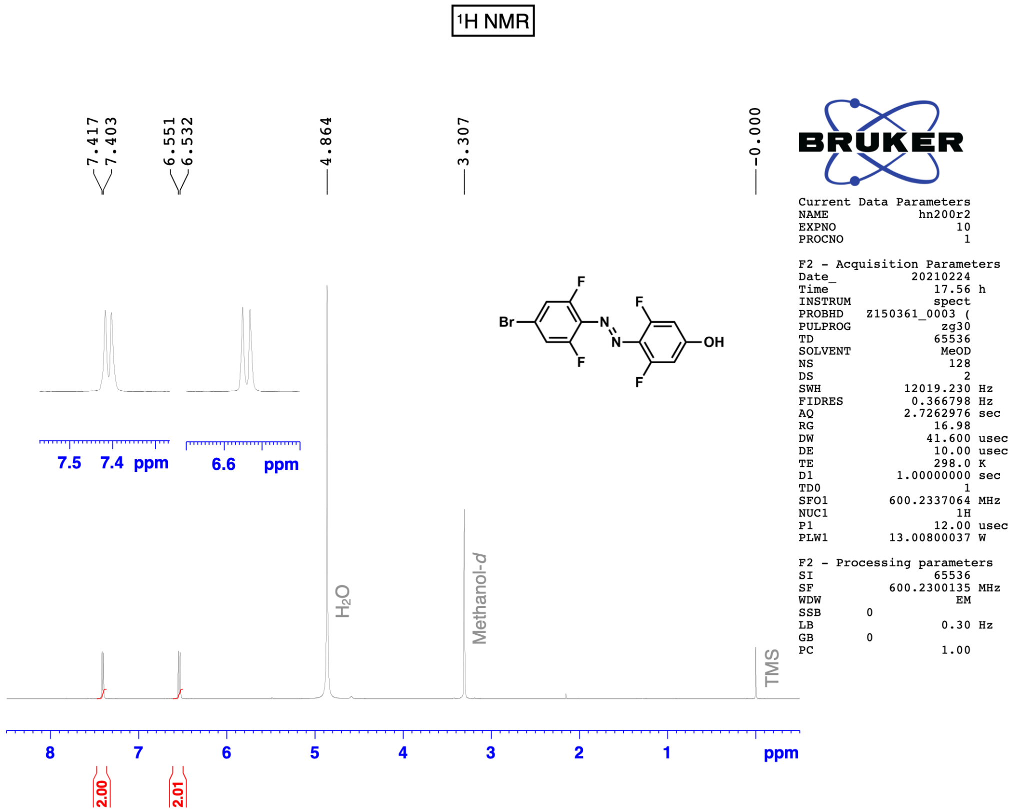

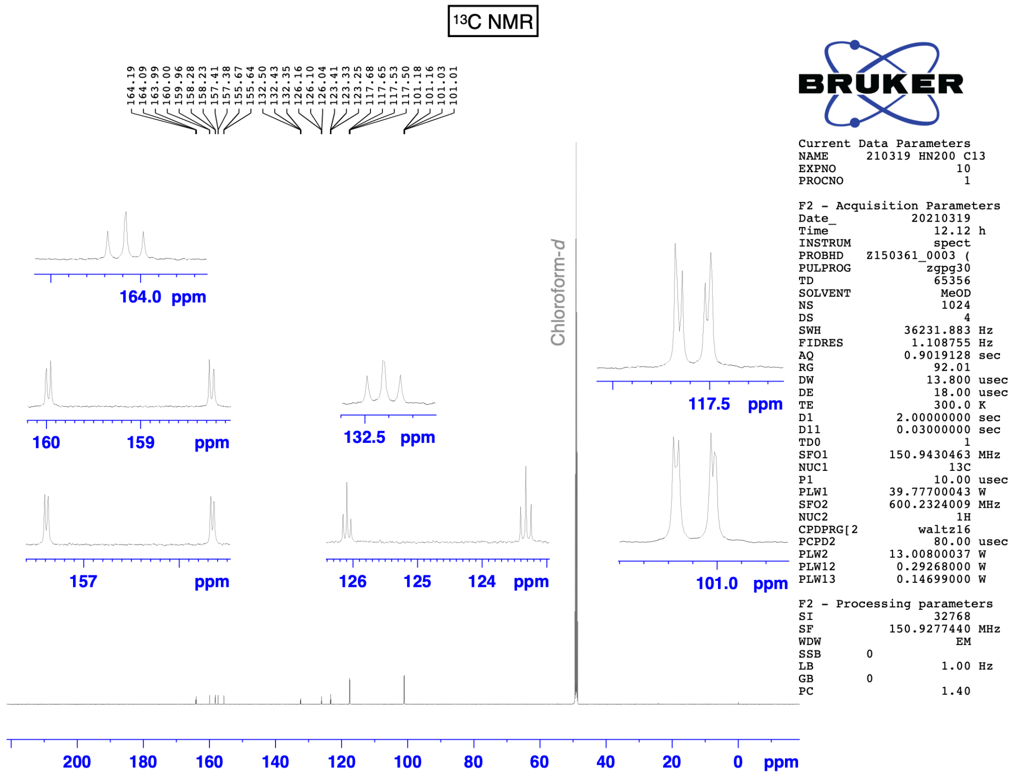

Supplementary Fig. 1 | ^{1}H NMR and ^{13}NMR spectra of compound 2.

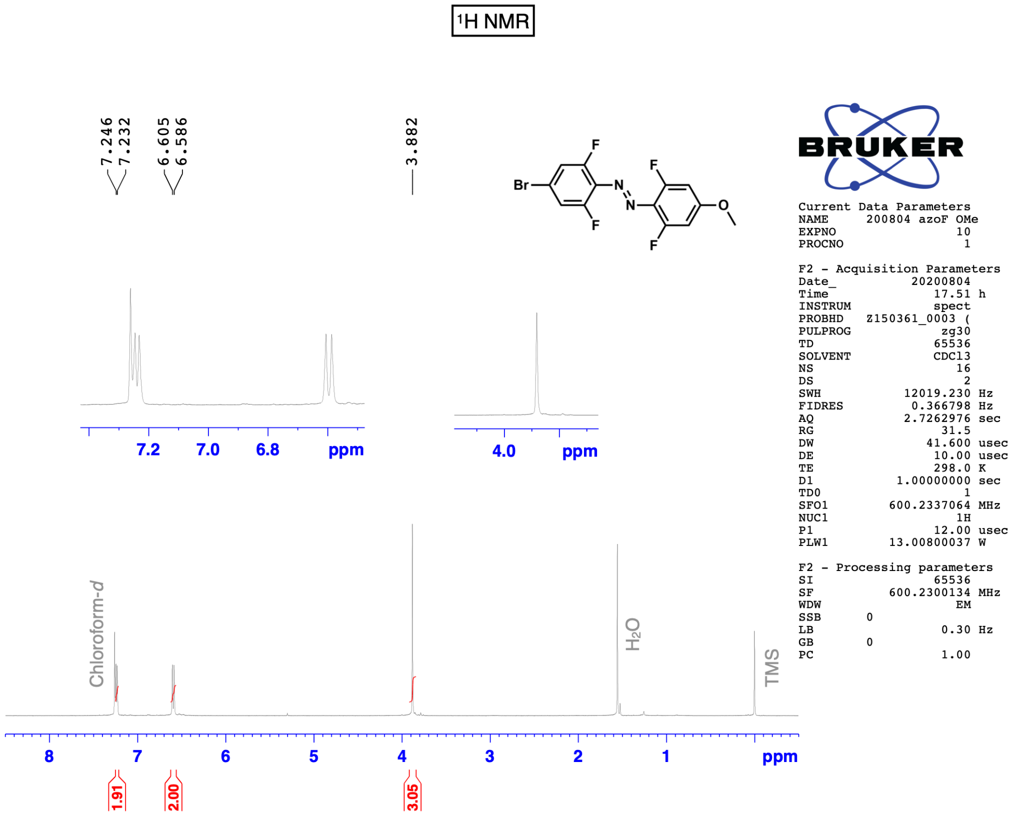

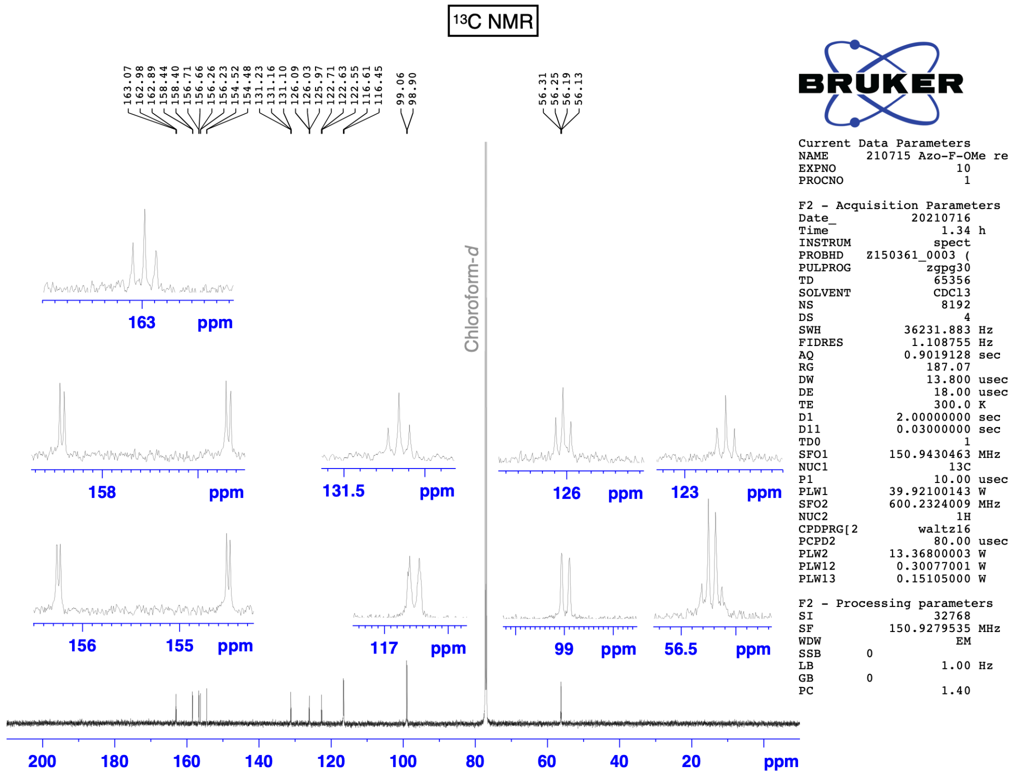

Supplementary Fig. 2 | ^{1}H NMR and ^{13}NMR spectra of compound 3.

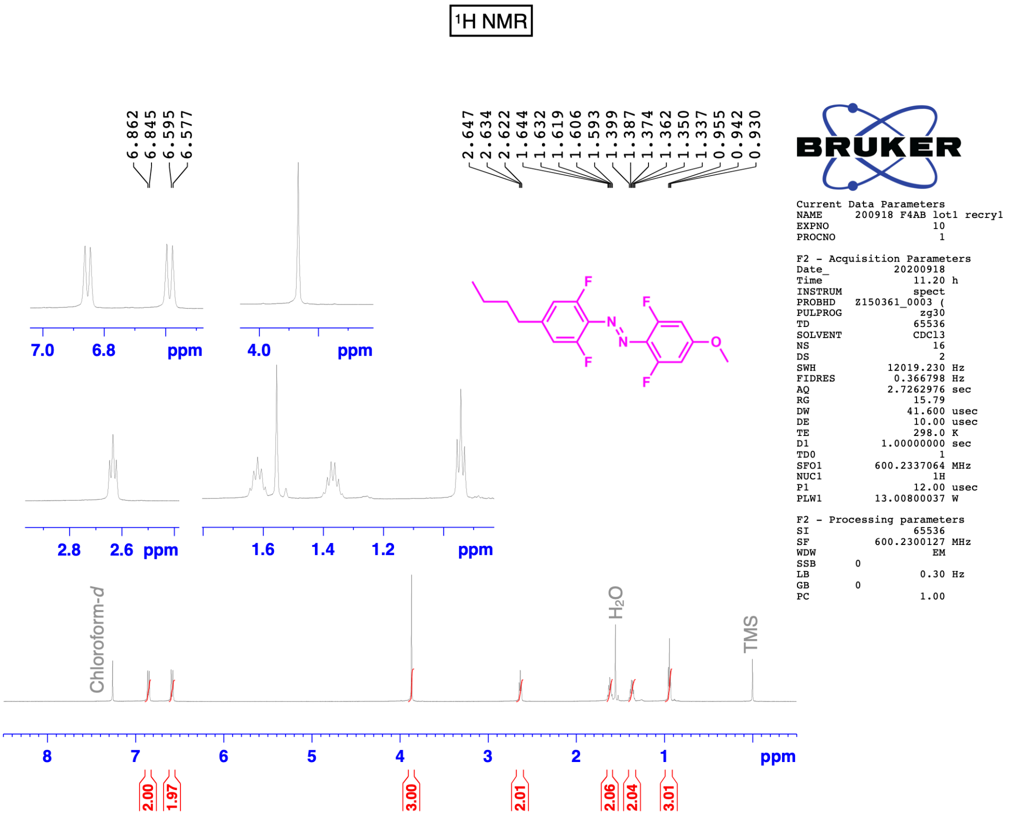

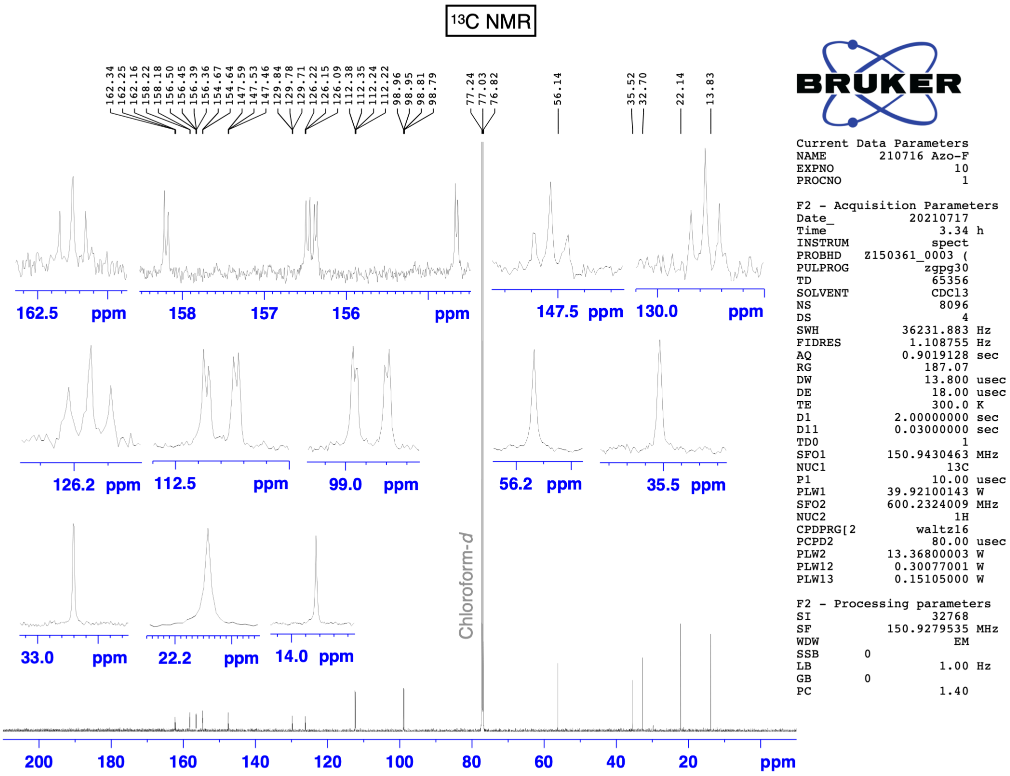

Supplementary Fig. 3 | ^{1}H NMR and ^{13}NMR spectra of compound 1 (Azo-F).

HRMS

[Mass Spectrum]
Data : Azo-F-OH-EI(+)LR   Date : 16-Aug-2021 14:13
Instrument : Station
Note : MStation : IMCE Kyushu Univ.
Ion Mode : EI+
Scan# : (30,40)
Cut Level : 1.00 %

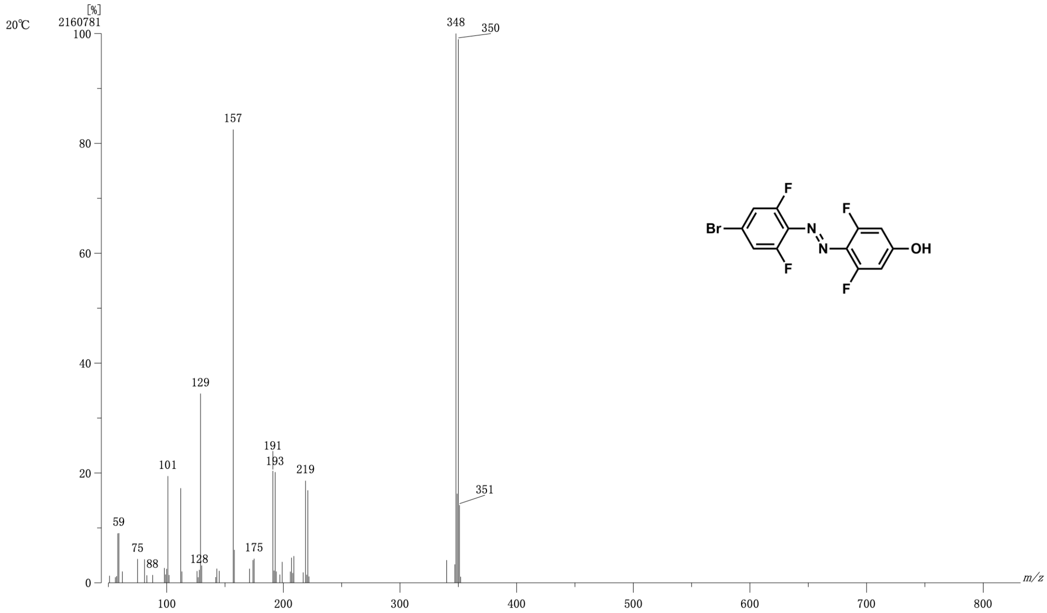

[Mass Spectrum]
Data : Azo-F-OH-EI(+)HR   Date : 16-Aug-2021 14:17
Instrument : Station
Note : MStation : IMCE Kyushu Univ.
Ion Mode : EI+
Scan# : (16,21)
Cut Level : 1.00 %
Internal standard:PFK

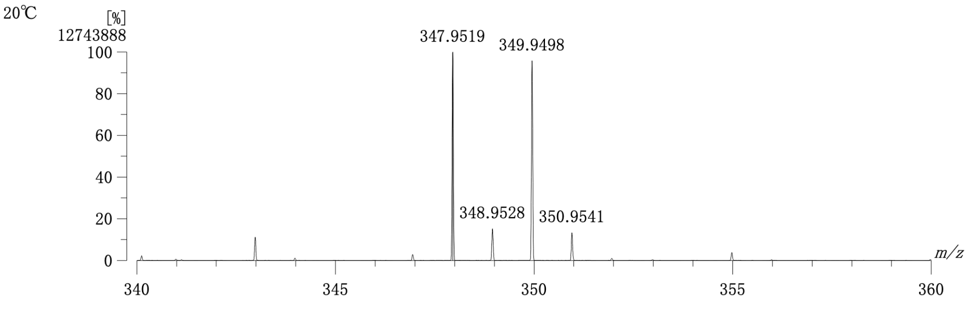

	Observed m/z	Int%	Err[ppm / mmu]	U.S.	Composition
1	347.9519	100.00	-0.7 / -0.2	9.0	C12 H5 Br F4 N2 O

Supplementary Fig. 4 | High-resolution mass spectra (HR-EIMS) of compound 2.

HRMS

[Mass Spectrum]
Data : Azo-F-OMe-EI(+)LR Date : 16-Aug-2021 14:42
Instrument : Station
Note : MStation : IMCE Kyushu Univ.
Ion Mode : EI+
Scan# : (20,30)
Cut Level : 1.00 %

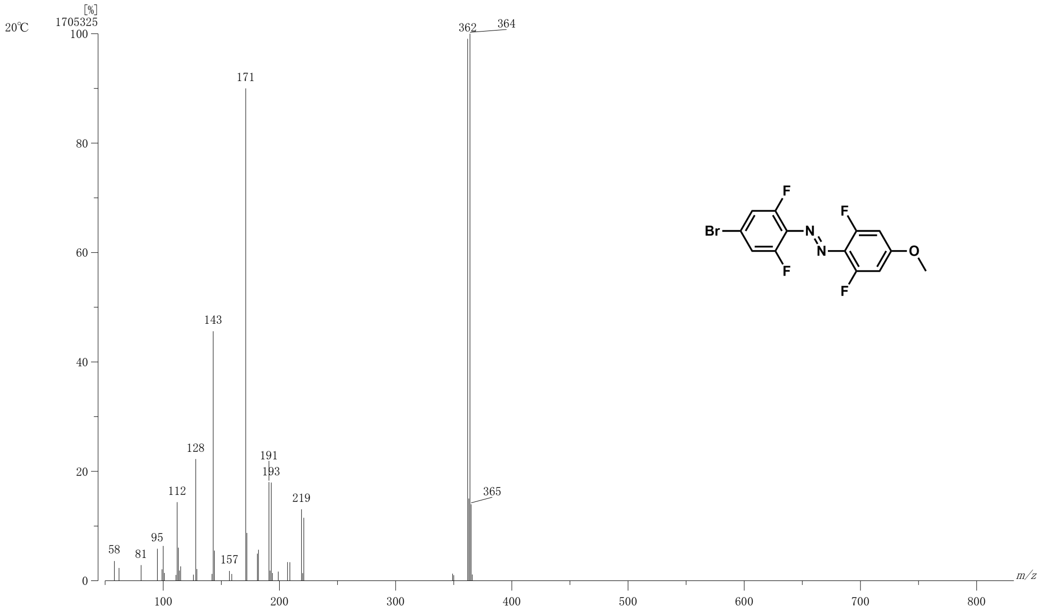

[Mass Spectrum]
Data : Azo-F-OMe-EI(+)HR Date : 16-Aug-2021 14:44
Instrument : Station
Note : MStation : IMCE Kyushu Univ.
Ion Mode : EI+
Scan# : (20,25)
Cut Level : 1.00 %
Internal standard:PFK

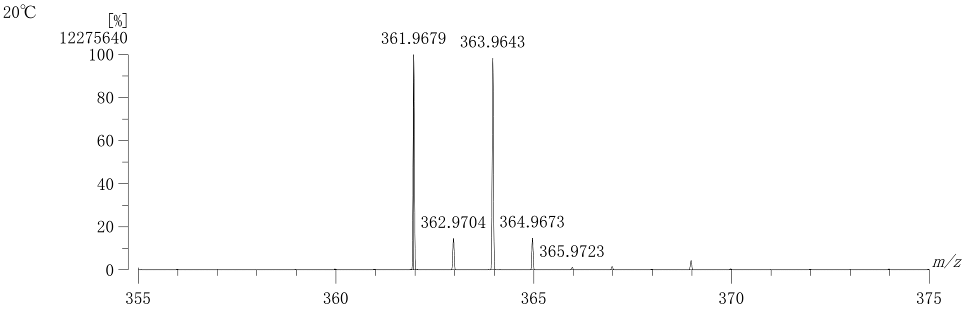

	Observed m/z	Int%	Err[ppm / mmu]	U.S.	Composition
1	361.9679	100.00	+0.3 / +0.1	9.0	C13 H7 Br F4 N2 O

Supplementary Fig. 5 | High-resolution mass spectra (HR-EIMS) of compound 3.

HRMS

[Mass Spectrum]
Data : Azo-F-EI(+)LR   Date : 16-Aug-2021 13:27
Instrument : Station
Note : MStation : IMCE Kyushu Univ.
Ion Mode : EI+
Scan# : (30,40)
Cut Level : 1.00 %

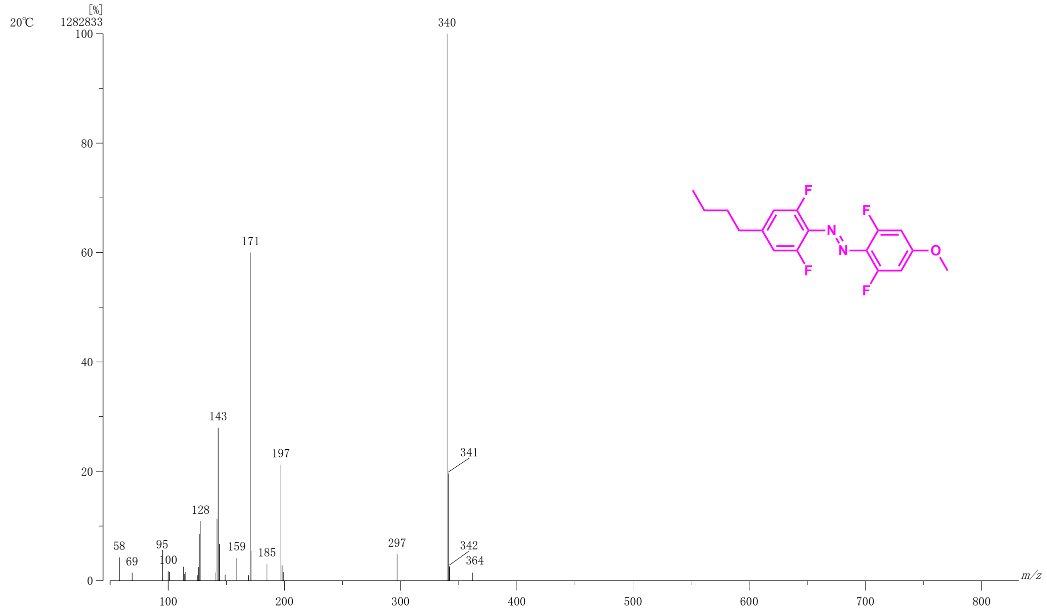

[Mass Spectrum]
Data : Azo-F-EI(+)HR   Date : 16-Aug-2021 13:30
Instrument : Station
Note : MStation : IMCE Kyushu Univ.
Ion Mode : EI+
Scan# : (20,24)
Cut Level : 1.00 %
Internal standard:PFK

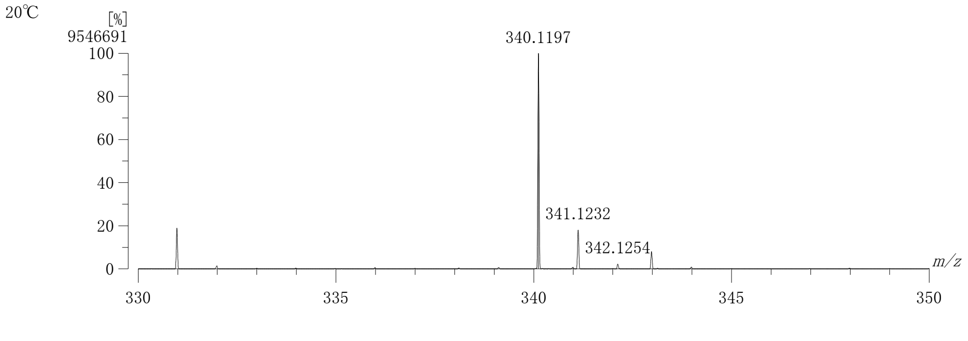

	Observed m/z	Int%	Err[ppm / mmu]	U.S.	Composition
1	340.1197	100.00	-0.5 / -0.2	9.0	C17 H16 F4 N2 O

Supplementary Fig. 6 | High-resolution mass spectra (HR-EIMS) of compound 1 (Azo-F).

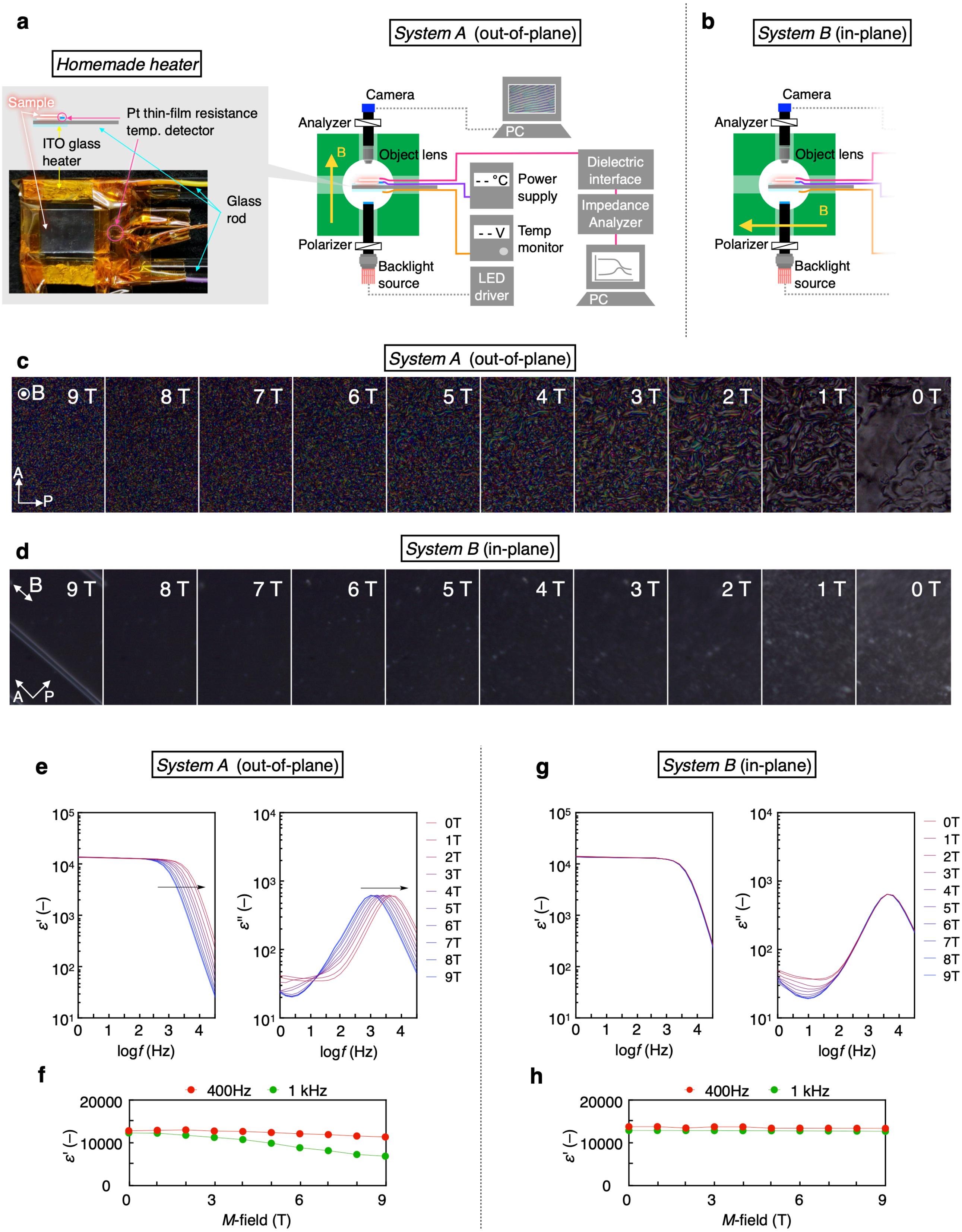

a
System A (out-of-plane)
Homemade heater
Sample
Pt thin-film resistance temp. detector
ITO glass heater
Glass rod
Camera
Analyzer
Object lens
Polarizer
Backlight source
PC
Power supply
Temp monitor
LED driver
Dielectric interface
Impedance Analyzer
b
System B (in-plane)
c
System A (out-of-plane)
9 T 8 T 7 T 6 T 5 T 4 T 3 T 2 T 1 T 0 T
d
System B (in-plane)
e
System A (out-of-plane)
ε' (–)
ε'' (–)
logf (Hz)
0T 1T 2T 3T 4T 5T 6T 7T 8T 9T
f
400Hz 1 kHz
M-field (T)
g
System B (in-plane)
h

Supplementary Fig. 7 | Gigantic dielectric properties of the ferronematic phase for DIO. Polarized optical microscopy and dielectric spectroscopy under a magnetic-filed in N_F phase (60 °C) of DIO (cell thickness: 21.4 μm, silanized). Measurement system under magnetic field vertical (a, system A) and parallel (b, system B) to the LC cell. A schematic illustration and actual image of a handmade hot-stage are shown in left side of the panel (a). Changes in polarized optical microscopy image in system A (c) and system B (d) under various applied magnetic field (9 T to 0 T). Dielectric properties (left: permittivity, ε' and right: loss, ε'') in out-of-plane (e) and in-plane system (f) under various applied magnetic field magnetic field (9 T to 0 T). Dielectric permittivity *vs*. applied magnetic field in out-of-plane (f) and in-plane system (h). Note: In this experiment, to keep devices away from strong magnetic field, we used long BNC cables (*ca*. 10 m) connecting an impedance analyzer and the LC cell, thereby sacrificing dielectric measurement in high frequency range ($\log f > 4.5$).

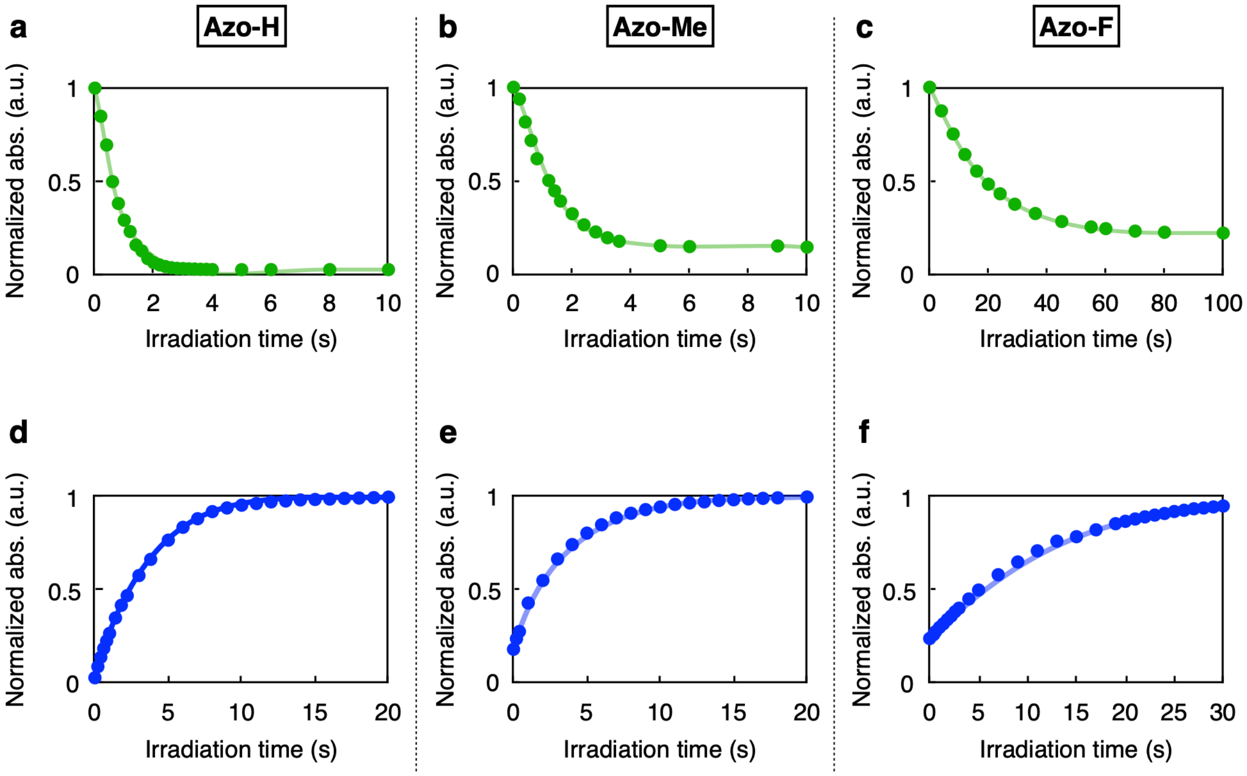

Supplementary Fig. 8 | Photo-isomerization of azobenzene-tethered phototriggers in MeCN.

Changes in the maximum absorbance of UV-Vis spectra (Fig. 1) at 350 nm (Azo-H), 336 nm (Azo-Me) and 335 nm (Azo-F) as a function of light irradiation time.

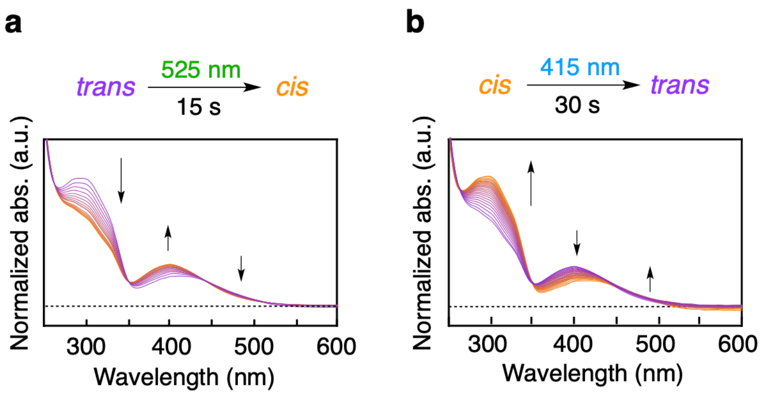

Supplementary Fig. 9 | Photo-isomerization of Azo-F in LC quartz cell.

Evolution of UV/Vis spectra of Azo-F (4 wt%) during alternate GL (**a**) and BL (**b**) irradiation. Fixed temperature = 52 °C.

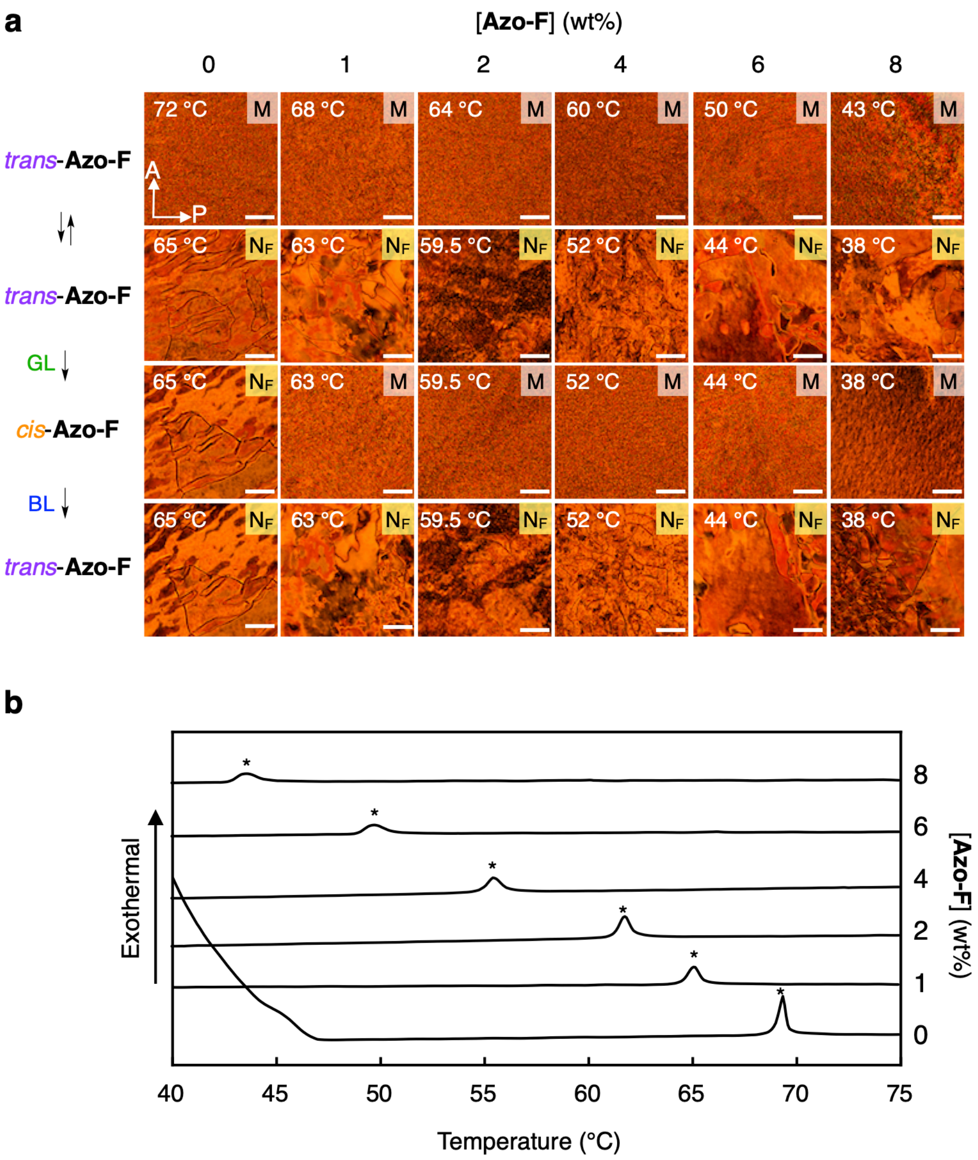

Supplementary Fig. 10 | Thermal properties of LC blends.

a, Changes in polarized optical microscopic images for LC blends ([Azo-F] = 0–8 wt%) taken through a long path filter ($\lambda > 550$ nm) under crossed polarizers in a LC cell (cell thickness: 17.8 μm, silanized). Photoisomerization was carried out within photo-controllable temperatures. **b**, DSC curves for LC blends ([Azo-F] = 0–8 wt%). Rate: 5 K min^{-1}. Asterisk denotes the M–N_F phase transition point. Scale bar: 100 μm.

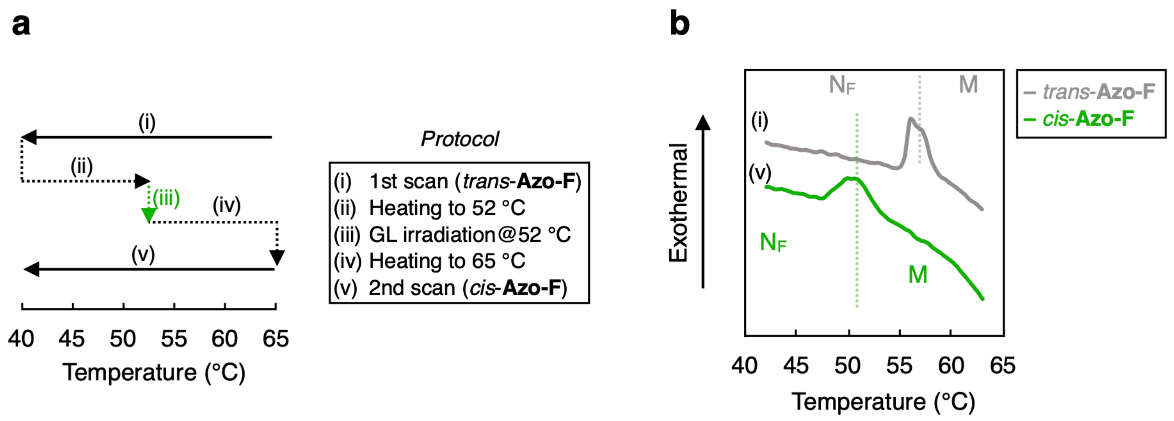

Supplementary Fig. 11 | DSC traces for a post-irradiated LC blend.

a, A protocol for DSC scanning. **b**, DSC curves for *trans* and *cis* states of a LC blend ([Azo-F] = 4 wt%) in the step of (i) and (v) indicated in the panel (**a**), respectively. In this experiment, we used the LC blend embedded in an aluminum crucible without a lid for the light irradiation so that the obtained curves were noisy; but the phase transition points were in good agreement with them observed in POM and dielectric studies.

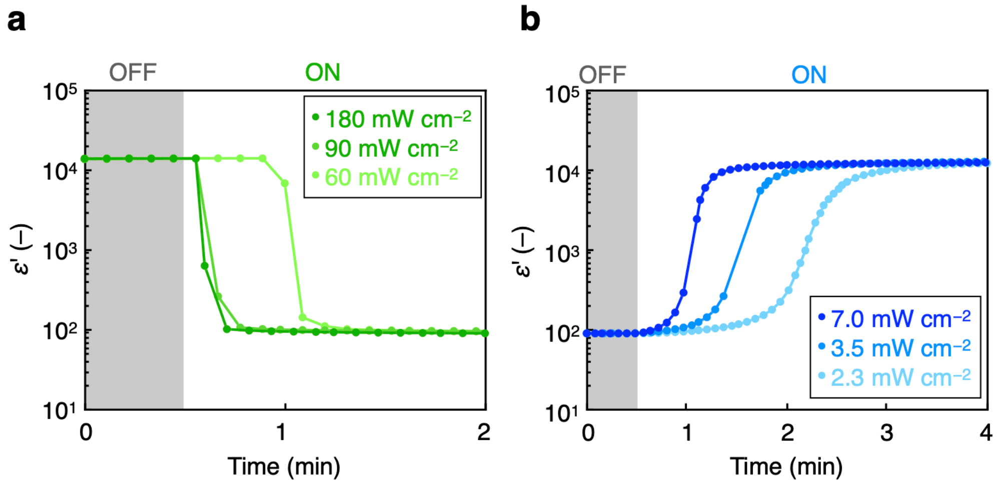

Supplementary Fig. 12 | Light power dependence of dielectric tunability.

Changes in dielectric permittivity ($f = 1$ kHz) for a LC blend ([Azo-F] = 4 wt%) upon GL irradiation (**a**) and BL irradiation (**b**). I_{GL} = 180, 90 and 60 mW cm^{-2}; I_{BL} = 7.0, 3.5 and 2.3 mW cm^{-2}. Fixed temperature = 53 °C.

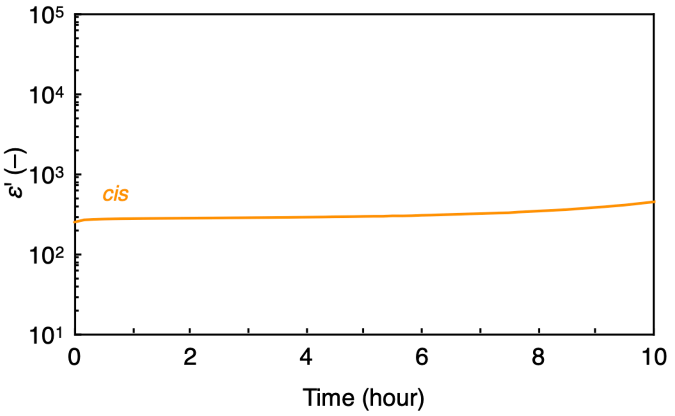

Supplementary Fig. 13 | Thermal relaxation test of *cis*-Azo-F.

The dielectric permittivity of a LC blend ([*cis*-Azo-F] = 4 wt%) at 52.0 °C was stable for 10 hours in dark. The *cis*-state was prepared by GL irradiation for 3 min and then kept in dark. It is known that the kinetics of the thermal back *cis*→*trans* reaction of azobenzene derivatives are strongly influenced by solvent environment (e.g. DMSO and LC) and temperature, so that the thermal relaxation behavior in this figure was different from that shown in Fig. 1j in the main text.

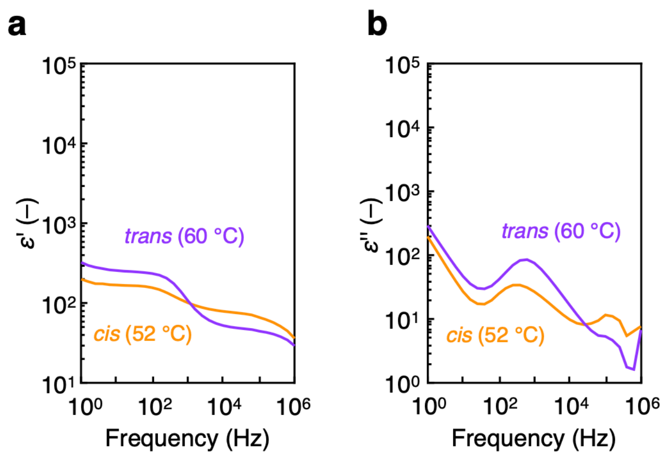

Supplementary Fig. 14 | Dielectric spectra of a LC blend ([Azo-F] = 4 wt%) in a LC cell in M phase at 60 °C and M phase exposed to GL at 52 °C.

a, Dielectric permittivity, **b**, dielectric loss. A purple-colored and orange-colored line denote dielectric spectra at 60 °C (*i.e.* [*trans*-Azo-F] = M phase) and at 52 °C (*i.e.* [*cis*-Azo-F] = M phase exposed to GL), respectively.

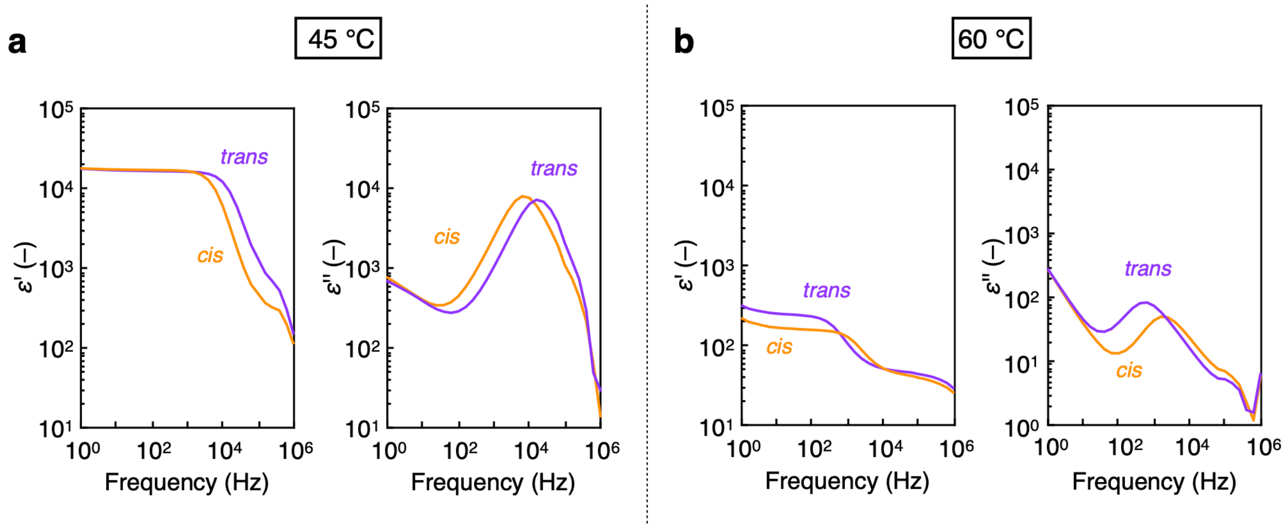

Supplementary Fig. 15 | Photo-tuning test of dielectric spectra of Azo-F in a LC cell.

Dielectric spectra of a LC blend ([Azo-F] = 4 wt%) in a LC cell before GL irradiation (purple-colored line), after GL irradiation (orange-colored line) at 45 °C (**a**) and 60 °C (**b**). There were no significant changes of dielectric spectra in out-of-range temperature for the photo-tuning.

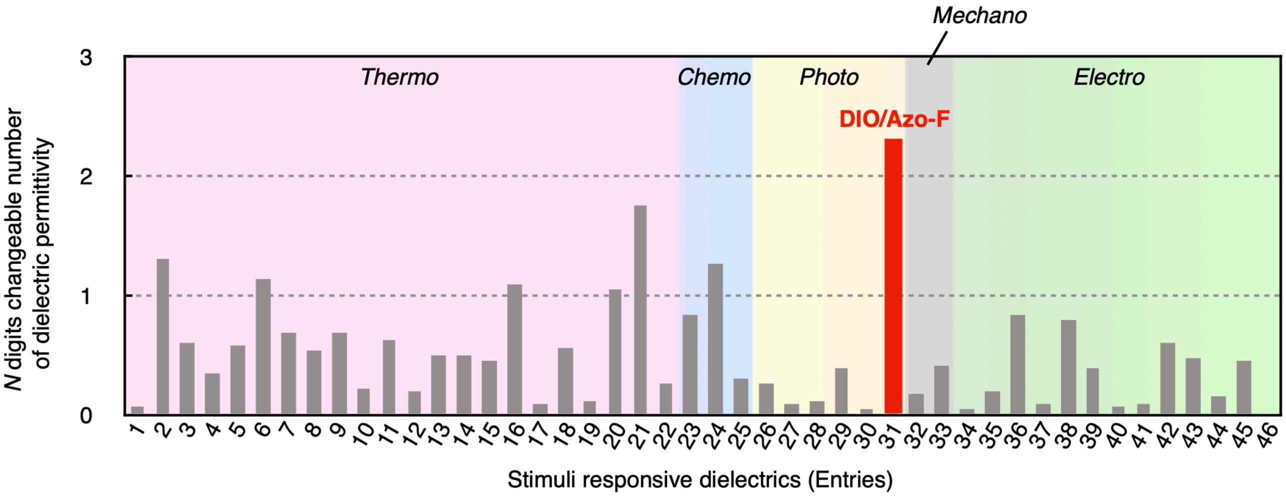

Supplementary Fig. 16 | Dielectric tunability of stimuli responsive dielectrics.

The entry and the corresponding materials are listed in Supplementary Table 1. A number of digits (N) was calculated by formula as follows: $N = \log\varepsilon'_{max} - \log\varepsilon'_{min}$.

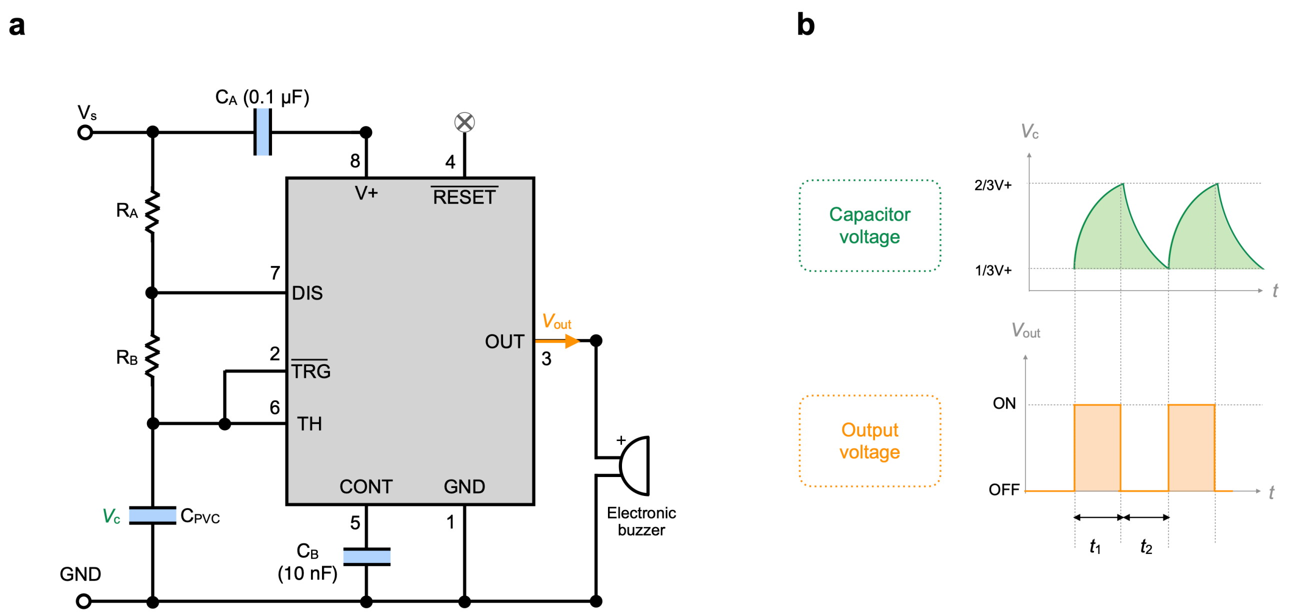

Supplementary Fig. 17 | Oscillator circuit.

a, GND, TRIG, OUT, CONT, THR, DIS and V+ stand for grand, trigger, output, control, threshold, discharge and supply voltage, respectively. In this experiment, we never used a RESET gate. *C* and *R* represent capacitor and resistor, respectively. C_A and C_B are the decoupling condenser (C_A = 0.1 μF; C_B = 10 nF). **b**, Output ON/OFF time periods (t_1 and t_2) determined by the suitable *RC* combinations.

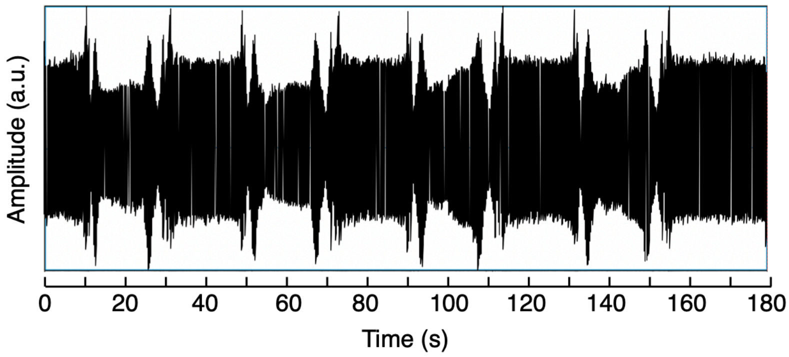

Supplementary Fig. 18 | Recorded audio wave.

Manipulation sequence during recording is as follows: dark (10 s) → GL irradiation (10 s) → dark (10 s) → BL irradiation (10 s) → ··· (totally four cycles). Sampling frequency: 44.1 kHz.

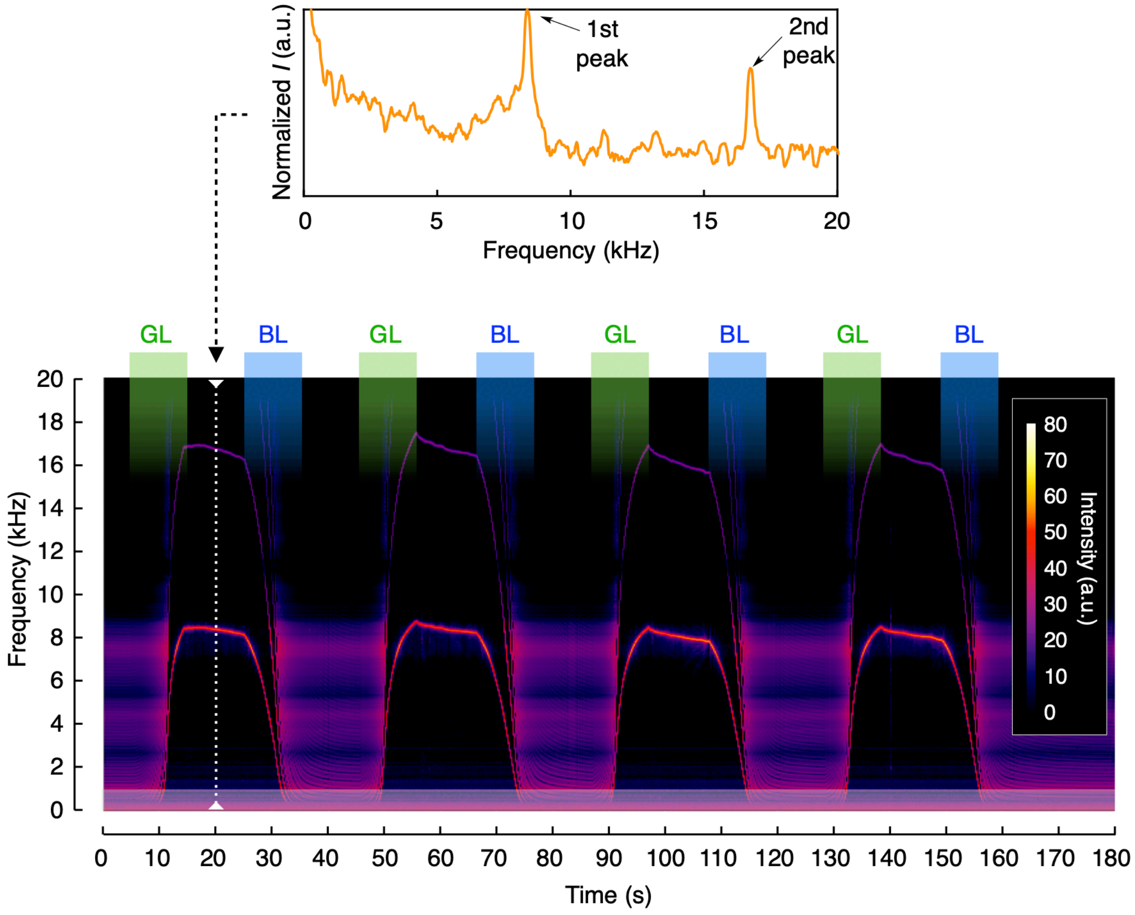

Supplementary Fig. 19 | Overall spectrogram of a LC blend ([Azo-F] = 4 wt%) during 4 cycles of alternation of GL/BL irradiation.

Upper panel denotes fundamental (1st) and overtone (2nd) peaks on the cross section indicated by white colored line at t = 20 sec.

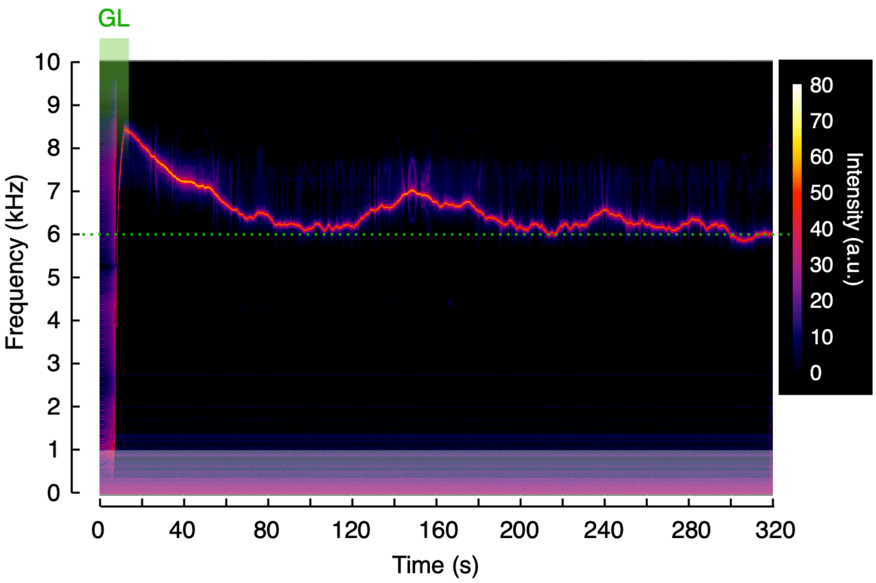

Supplementary Fig. 20 | Spectrogram of a LC blend ([Azo-F] = 4 wt%) after GL irradiation (10 sec) followed by keeping (5 min) in dark.

Supplementary Table 1 | Common dielectric tunability.

Stimuli	Entry	Materials	Dielectric permittivity ε_{min} / ε_{max} / $\Delta\varepsilon$			Relative tunability [%]	Measuring frequency [Hz]	Ref.
Thermo	1	$[C_3H_5NH_3]_2[CdCl_4]$ [c)]	10.6	12.3	13.8	1.7	200 k	4
	2	$[C_6H_{12}NH_3][PbBr_4]$ [c)]	20	400	380	95	100 k	5
	3	$(MA)_2[B'B''(CN)_6]$ [c)]	6	24	18	75	1 M	6
	4	$(H_2hpz)[K(ClO_4)_3]$ [c)]	8.4	18.3	9.9	54	100 k	7
	5	$(HIm)_2[KCo(CN)_6]$ [c)]	6	23	17	74	1 M	8
	6	N-isopropylbenzylammonium tetrafluoroborate [c)]	10	136	126	88	5 k	9
	7	Coordination polymer $[(CH_3)_2NH_2]$ $[Cd(N_3)_3]$ [c)]	2.7	13.2	10.5	80	1 M	10
	8	$(MA)(H_2O)[Sr(H_2O)_2Co(CN)_6]$ [c)]	7	24	17	71	1 M	11
	9	$[(CH3)_2NH_2]^{2-}$ $[KCo(CN)_6]$ [c)]	4	19	15	79	1 M	12
	10	$[(CH_3CH_2)_2NH_2]$ $[Fe^{III}Fe^{II}(HCOO)_6]$ [c)]	11	18	7	39	1 M	13
	11	$(HIm)_2[KFe(CN)_6]$ [c)]	6	25	19	76	1 M	14
	12	$(Me_3NH)_4[Ni(NCS)_6]$ [c)]	4.2	6.7	2.3	34	1 M	15
	13	$[(C_5H_{10})(CH_3)NH][18\text{-crown-}6][ClO_4]\cdot H_2O$ [a)]	1.2	3.7	2.5	68	1 M	16
	14	bis(2-chloroethyl)amine hydrochloride [a)]	5	14.6	9.6	68	1 M	17
	15	bis(2-chloroethyl)amine hydrochloride [a)]	6	17	11	65	10 k	17
	16	(*R*)-(–)-3-hydroxlyquinuclidinium halide [a)]	5	62	57	92	1 M	18
	17	Di-*n*-butylaminium trifluoroacetate [a)]	7.8	9.5	1.7	18	100 k	19
	18	Allyl-trimethyl-phosphonium [a)]	5	18	13	72	1 M	20
	19	[trimethylallyl ammonium]$_3[Bi_2Cl_9]$ [a)]	4.77	6.30	1.53	24	1 M	21
	20	Imidazolium periodate (IPI) [a)]	10	110	100	91	1 M	22
	21	ODA-MWCNTs/hexadecane composites (2 vol%) [c)]	9.9	550.3	541.3	98	1	23
	22	PVDF/PEG/GO composites, 16 wt% (PEG/GO) [c)]	34.6	61.5	26.9	44	100	24
Chemo	23	$(MA)(H_2O)[Sr(H_2O)_2Co(CN)_6]$	3.5	24	20.5	85	1 M	11
	24	H2O-HKUST1 (MOF)	3	54	51	94	1 M	25
	25	MeOH-HKUST1 (MOF)	5	10	5	50	1 M	25

[a)] O: organic material, [b)] I: inorganic material, [c)] O/I: organic and inorganic hybrid.

Supplementary Table 1 | Common dielectric tunability (continued).

Stimuli	Entry	Materials	Dielectric permittivity ε_{min} / ε_{max} / $\Delta\varepsilon$			Relative tunability [%]	Measuring frequency [Hz]	Ref.
Photo	26	$LaAl0_{.99}Zn_{0.01}O_{3-\delta}$ [b)]	25	45	20	44	100	26
	27	$Ba(Al_{0.97}Zn_{0.03})_2O_{4-\delta}$ [b)]	17	21	4	19	1 M	27
	28	$Ba(Al_{0.95}Zn_{0.05})_2O_{4-\delta}$ [b)]	16	23	5	22	1 M	28
	29	Salicylideneaniline [a)]	20	50	30	60	100 k	29
	30	$\{[Zn_2(3\text{-bpep})_2(2,5\text{-FDC})_2]\cdot 1.5H_2O\}_n$ [c)]	4.1	4.6	0.5	11	1	30
	31	**DIO / Azo-F** (4 wt%) [a)]	91	1.8k	1.79k	99.5	1 k	Our work
Mechano	32	ZrO_2/Ge film (tension)	18	27.5	9.5	35	N/A	31
	33	ZrO_2/Ge film (compression)	7	18	11	61	N/A	31
Electro	34	BTO [b)]	4.5	5	0.5	10	300 T	32
	35	STO [b)]	264	424	160	38	5.3 G	33
	36	$Pb(Sc_{1/2}Ta_{1/2})O_3$ (PST) [b)]	3k	21k	18k	88	1 k	34
	37	$(Ba,Sr)TiO_3$ (BST) [b)]	N/A	3.8k	760	80	10–30 G	35
	38	Al_2O_3-BST [b)]	N/A	870	732	16	7.7 G	36
	39	Mg-BST (3 mol% Mg) [b)]	N/A	339	203	40	100 k	37
	40	$Ba(Zr,Ti)O_3$ (BZT), $Ba(Zr_{0.2}Ti_{0.8})O_3$ [b)]	N/A	7.6k	1.1k	86	10 k	38
	41	$Pb(Mg_{1/3}Nb_{2/3})O_3PbTiO_3$(0.76PMN-0.24PT) [b)]	N/A	30k	6k	80	1 k	39
	42	$Pb(Mg_{1/3}Nb_{2/3})O_3PbTiO_3$ (0.76PMN-0.24PT) [b)]	N/A	40k	30k	25	1 k	39
	43	$PbZrO_3$ [b)]	N/A	419	279	33	100 k	40
	44	W-$PbZrO_3$ (5% W) [b)]	N/A	1.4k	418	70	1 k	41
	45	$[NH_2(CH_3)_2]_n[Fe^{III}Fe^{II}(HCOO)_6]_n$	800	1.2k	780	35	100	42
	46	P(VDF-TrFE)	4.42	4.62	0.2	4	2 G	43

[a)] O: organic material, [b)] I: inorganic material, [c)] O/I: organic and inorganic hybrid.

Supplementary Table 2 | Thermal properties of [Azo-F] (0–8 wt%)

Conc. [wt%]	M-N$_F$ phase transition Temp [°C] [a)] DR	DSC	$\Delta H_{M\text{–}NF}$ [kJ mol^{-1}]	$\Delta H_{estimated}$ [kJ mol^{-1}] [b)]
0	68.0	69.4	0.199	-
1	64.5	65.1	0.193	0.198
2	61.6	61.8	0.193	0.196
4	55.1	55.4	0.191	0.192
6	48.5	49.8	0.125	0.188
8	40.2	43.6	0.099	0.184

[a)] M–N$_F$ phase transition temperature detected by dielectric relaxation spectroscopy (Fig. 2a in the main text) and DSC (Supplementary Fig. 10b), [b)] an estimated ΔH was calculated by the formula as follows: $\Delta H_{estimated} = \phi \cdot \Delta H_{M-N_F}^{pure\ DIO}$, where ϕ and $\Delta H_{M-N_F}^{pure\ DIO}$ denote a volume function of Azo-F and of the enthalpy due to the M–N$_F$ phase transition of pure DIO, respectively.

Supplementary Audio

Supplementary Audio 1 | A change in the pitch of a recorded audio.
The audio from the electric speaker was recorded using a microphone. Manipulation sequence during recording is as follows: dark (10 s) → GL irradiation (10 s) → dark (10 s) → BL irradiation (10 s) → ··· (totally four cycles). Sampling frequency: 44.1 kHz.

Supplementary References

1. Tazuke, S., Kurihara, S. & Ikeda, T. Amplified image recording in liquid crystal media by means of photochemically triggered phase transition. *Chem. Lett.* 911–914 (1986).

2. Tsai, B.-K. et al. Photoswitchable fluorescence on/off behavior between *cis*- and *trans*-rich azobenzenes. *J. Mater. Chem. C* **22**, 20874–20877 (2012).

3. Contreras, A., Garcia-Azpeitia, C., García-Cervera, C. J. & Joo, S. The onset of layer undulations in smectic A liquid crystals due to a strong magnetic field. *Nonlinearity* **29**, 2474–2496 (2016).

4. Han, S.et al. Dielectric phase transition triggered by the order–disorder transformation of cyclopropylamine in a layered organic–inorganic halide perovskite. *J. Mater. Chem. C* **6**, 10327–10331 (2018).

5. Sun, Z. et al. A photoferroelectric perovskite-type organometallic halide with exceptional anisotropy of bulk photovoltaic effects. *Angew. Chem. Int. Ed.* **55**, 6545–6550 (2016).

6. Shi, C., Yu, C.-H. & Zhang, W. Predicting and screening dielectric transitions in a series of hybrid organic–inorganic double perovskites via an extended tolerance factor approach. *Angew. Chem. Int. Ed.* **55**, 5798–5802 (2016).

7. Sun, Y.-L., Shi, C. & Zhang, W. Distinct room-temperature dielectric transition in a perchlorate-based organic–inorganic hybrid perovskite. *Dalton. Trans.* **46**, 16774–16778 (2017).

8. Zhang, X et al. Dynamics of a caged imidazolium cation–toward understanding the order-disorder phase transition and the switchable dielectric constant. *Chem. Commun.* **51**, 4568–4571 (2015).

9. Ji, C. et al. N-Isopropylbenzylammonium tetrafluoroborate: an organic dielectric relaxor with a tunable transition between high and low dielectric states. *J. Mater. Chem. C* **2**, 567–572 (2014).

10. Du, Z.-Y. et al. Switchable guest molecular dynamics in a perovskite-like coordination polymer toward sensitive thermoresponsive dielectric materials. *Angew. Chem. Int. Ed.* **54**, 914–918 (2015).

11. Lu, Y.-L. & Zhang, W. Dual stimuli-triggered dielectric switching and sensing in a host–guest cyanometallate framework. *Chem. Commun.* **53**, 6077–6080 (2017).

12. Zhang, W. et al. Tunable and switchable dielectric constant in an amphidynamic crystal. *J. Am. Chem. Soc.* **135**, 5230–5233 (2013).

13. Zhao, J.-P. et al. A Niccolite structural multiferroic metal–organic framework possessing four different types of bistability in response to dielectric and magnetic modulation. *Adv. Mater.* **29**, 1606966 (2017).

14. Zhang, W. et al. Exceptional dielectric phase transitions in a perovskite-type cage compound. *Angew. Chem., Int. Ed.* **49**, 6608–6610 (2010).

15. Liu, J.-Y. et al. Molecular dynamics, phase transition and frequency-tuned dielectric switch of an ionic co-crystal. *Angew. Chem., Int. Ed.* **57**, 8032–8036 (2018).

16. Khan, T. et al. A supra-molecular switchable dielectric material with non-linear optical properties. *J. Mater. Chem. C* **5**, 2865–2870 (2017).

17. Shao, X.-D. et al. Switching dielectric constant near room temperature in a molecular crystal. *Adv. Sci.* **2**, 1500029 (2015).

18. Li, P.-F. et al. Anomalously rotary polarization discovered in homochiral organic ferroelectrics. *Nat. Commun.* **7**, 13635 (2016).

19. Sun, Z. et al. Ultrahigh pyroelectric figures of merit associated with distinct bistable dielectric phase transition in a new molecular compound: di-n-butylaminium trifluoroacetate. *Adv. Mater.* **27**, 4795 (2015).

20. Zhou, L. et al. Molecular design of high-temperature organic dielectric switches. *Chem. Commun.* **54**, 13111–13114 (2018).

21. Wang, Q. et al. A high-temperature multiaxial precision time-delayed dielectric switch crystal triggered by linear/propeller/ball three-form motion. *J. Mater. Chem. C* **7**, 2994–3002 (2019).

22. Zhang, Y. et al. Switchable dielectric, piezoelectric, and second-harmonic generation bistability in a new improper ferroelectric above room temperature. *Adv. Mater.* **26**, 4515 (2014).

23. Meng, P. et al. Room-temperature dielectric switchable nanocomposites. *Adv. Funct. Mater.* **27**, 1701136 (2017).

24. Tu, J. et al. Phase change-induced tunable dielectric permittivity of poly(vinylidene fluoride)/polyethylene glycol/graphene oxide composites. *Composites Part B*, **173**, 106920 (2019).

25. Babal, A. S., Chaudhari, A. K., Yeung, H. H.-M. & Tan, J.-C. Guest-tunable dielectric sensing using a single crystal of HKUST-1. *Adv. Mater. Interfaces* **7**, 2000408 (2020).

26. Nagai, T. et al. Optical control of dielectric permittivity in $LaAl_{0.99}Zn_{0.01}O_{3-\delta}$. *Appl. Phys. Lett.* **110**, 172901 (2017).

27. Nagai, T. et al. Photo-induced persistent enhancement of dielectric permittivity in $Zn:BaAl_2O_4$. *Appl. Phys. Lett.* **111**, 232902 (2017).

28. Nagai, T., Tanabe, K., Terasaki, I. & Taniguchi, H. Systematic tuning of the photo-dielectric effect in $Ba(Al_{1-x}Zn_x)_2O_{4-\delta}$. *Appl. Phys. Lett.* **113**, 212902 (2018).

29. Shimayama, K. et al. Photoswitching of the dielectric property of salicylideneaniline. *J. Incl. Phenom. Macrocycl. Chem.* **82**, 219 (2015).

30. Sima, J.-Y. et al. Reversible dielectric switching behavior of a 1D coordination polymer induced by photo and thermal irradiation. *Chem. Commun.* **55**, 3532–3535 (2019).

31. Narayanachari, K. V. L. V. et al. Growth stress induced tunability of dielectric permittivity in thin films. *J. Appl. Phys.* **119**, 014106 (2016).

32. Misra, S., Kalaswad, M., Zhang, D. & Wang, H. Dynamic tuning of dielectric permittivity in $BaTiO_3$ via electrical biasing. *Mater. Rec. Lett.* **8**, 321–327 (2020).

33. Adam, M., Fuchs D. & Schneider, R. $YBa_2Cu_3O_7$ microwave resonator tuned by epitaxial $SrTiO_3$ thin films. *Physica C* **372**, 504–507 (2002).

34. Iwata, M., Tamaoki, N., Arimoto, Y. & Ishibashi, Y. Dielectric tunability in $Pb(Sc_{1/2}Ta_{1/2})O_3$ single crystals. *Jpn. J. Appl. Phys.* **56**, 10PB01 (2017).

35. Yuan, Z. et al. Large dielectric tunability and microwave properties of Mn-doped $(Ba, Sr)TiO_3$ thin films. *Appl. Phys. Lett.* **87**, 152901 (2005).

36. Chong, K. B. et al. Improvement of dielectric loss tangent of Al_2O_3 doped $Ba_{0.5}Sr_{0.5}TiO_3$ thin films for tunable microwave devices. *J. Appl. Phys.* **95**, 1416 (2004).

37. Cole, M. W. et al. Low dielectric loss and enhanced tunability of $Ba_{0.6}Sr_{0.4}TiO_3$ based thin films via material compositional design and optimized film processing methods. *J. Appl. Phys.* **93**, 9218 (2003).

38. Tang, X. G., Chew, K.-H. & Chan, H. L. W. Diffuse phase transition and dielectric tunability of $Ba(Zr_yTi_{1-y})O_3$ relaxor ferroelectric ceramics. *Acta Materialia* **52**, 5177–5183 (2004).

39. Lu, S. G., Xu, Z. & Chen, H., Field-induced dielectric singularity, critical exponents, and high-dielectric tunability in [111]-oriented $(1-x)Pb(Mg_{1/3}Nb_{2/3})O_{3-x}PbTiO_3$ (x=0.24). *Phys. Rev. B* **72**, 054120 (2005).

40. Liu, Y. et al. Tunable electric properties of $PbZrO_3$ films related to the coexistence of ferroelectricity and antiferroelectricity at room temperature. *Appl. Phys. Lett.* **100**, 212902 (2012).

41. Sa, T., Qin, N., Yang, G. & Bao, D. W-doping induced antiferroelectric to ferroelectric phase transition in $PbZrO_3$ thin films prepared by chemical solution deposition. *Appl. Phys. Lett.* **102**, 172906 (2013).

42. Guo, J.-B. et al. Dielectric tunability, expanding the function of metal-organic frameworks. *Phys. Status Solidi RRL* 1700425 (2018).

43. Han, S.-W. et al. Investigation of frequency-dependent permittivity tunability of P(VDF-TrFE)metal-ferroelectric-metal capacitor. *Results Phys.* **12**, 469–470 (2019).
