# Supplementary material for: Anisotropic fluid with phototunable dielectric permittivity
Source: Nat Commun. 2022 Mar 3;13:1142. doi: 10.1038/s41467-022-28763-1 (PMC8894468; doi:10.1038/s41467-022-28763-1)
Supplement: Supplementary file 3 — Description to Supplementary Information [file 41467_2022_28763_MOESM3_ESM.docx]

**Supplementary Audio**

**Supplementary Audio 1** | **A change in the pitch of a recorded audio**.

The audio from the electric speaker was recorded using a microphone. Manipulation sequence during recording is as follows: dark (10 s) → GL irradiation (10 s) → dark (10 s) → BL irradiation (10 s) → ∙∙∙ (totally four cycles). Sampling frequency: 44.1 kHz.
